# Effect of attentional bias modification on pre-competition anxiety in athletes

**DOI:** 10.3389/fpsyg.2025.1596298

**Published:** 2025-08-21

**Authors:** Jing Zhao, Yuhan Yang, Heng Zhang, Yu Nie, Qiulin Wang

**Affiliations:** ^1^Harbin Institute of Technology, Haerbin, China; ^2^College of Physical Education, Yangzhou University, Yangzhou, Jiangsu, China

**Keywords:** attentional bias correction, attentional bias, athletes, pre-game anxiety, basketball player

## Abstract

**Background:**

Under high-pressure situations, such as crucial games, some athletes often underperform. This is the case even for exceptional athletes in critical moments of competition. Athletes often experience performance anxiety, which creates attentional errors and underperformance. Attentional bias, where emotional stimuli influence decision-making, may also result in selective attention to negative information. Attentional bias correction training (ABMT) aims to modify these attention patterns with the aim of alleviating anxiety symptoms. In this study, we investigated the pre-competition attention patterns among high-level athletes to enhance performance.

**Methods:**

Attentional bias correction training was employed to train 32 athletes for 4 weeks, every 2 days (emotional pictures for the experimental group and star pictures for the control group). A point detection paradigm was also utilized to test the athletes’ attentional bias behavior in a pre-competition anxiety situation before and after training.

**Results:**

The results revealed a significant main effect of test time on self-rated anxiety level of high-level athletes (*F*(1, 32) = 204.072, *p* < 0.001, η^2^p = 0.919), while the main effect of group was not significant (*F*(1, 32) = 0.505, *p* > 0.05, η^2^p = 0.025). Moreover, significant interaction was recorded between the test time and group (*F*(1, 32) = 124.895, *p* < 0.001, η^2^p = 0.874). In the simple effects analysis, high-level athletes in the experimental group had significantly lower pre-competition anxiety scores at the post-test than at the pre-test (43.90 ± 2.57 vs. 57.00 ± 2.77, *p* < 0.001). However, for the control group, there was no significant difference between the two groups (52.20 ± 2.57 vs. 53.80 ± 2.77, *p* = 0.133). In addition, the attentional bias scores were significantly different before and after the training (5.985 ± 1.045 vs. -0.613 ± 0.60, *p* < 0.05) in the experimental group, whereas there were no significant differences in the control group (7.813 ± 1.045 vs. 5.773 ± 0.613, *p* > 0.05).

**Conclusion:**

The present findings demonstrate that attentional bias correction training can effectively reduce pre-match anxiety and attentional bias toward negative information in high-level athletes. These results provide an important foundation for enhancing pre-competition attention training and mood regulation in athletes. Future research should explore the underlying mechanisms and practical applications of these findings to facilitate the development of strategies to improve pre-match attention training.

## Introduction

One of the distinguishing characteristics of competitive sports, basketball, requires the precise execution of complex maneuvers in an environment that is highly pressurized and subject to extensive social evaluation (Mehrsafar et al., 2020). Athletes’ emotional fluctuations may lead to impaired concentration, delayed reactions, and poor decision-making, ultimately reducing performance (Beilock et al., 2002; Lewis and Linder, 1997). Therefore, athletes not only need to have outstanding technical and tactical levels, but also need to employ effective coping strategies to manage stress and maintain optimal performance. In the process of competition, when facing pressure, the individual’s judgment and decision-making ability will often be affected, and it is easy to produce anxiety and other negative emotions (Lv et al., 2018). This psychological state will make athletes pay too much attention to the negative stimuli, creating a self-perpetuating pattern that impairs performance and increases the likelihood of losing the game, which will eventually lead to the loss of the game, making it difficult for them to perform at their best competitive level (Wang, 2018).

The underperformance observed under high-pressure situations is due to factors, such as distraction, delayed reaction, and overreaction. Attention theory refers to how the allocation of cognitive resources is altered in anxious situations (Amir et al., 2009; Bar-Haim et al., 2007; Beilock et al., 2002; Lewis and Linder, 1997). For example, anxious athletes may direct cognitive resources toward anticipated failure, reducing capacity for in-the-moment decision-making. Being an important internal psychological mechanism through which individuals process cognitive information, attention is a highly selective. Attentional bias occurs when the emotional meaning of a stimulus influences the individual’s decision-making capacity (Bar-Haim et al., 2007). Attentional bias is defined as situations in which individuals are highly sensitive to specific stimuli and show selective attention when perceiving and processing information (Wieckowski et al., 2019). People tend to prioritize attention to threatening stimuli compared to neutral stimuli and respond by converging or avoiding them (Bar-Haim et al., 2007). These findings highlight the importance of modifying attentional patterns to counter the predisposition to focus on threatening stimuli.

Attention bias modification training (ABMT) is a procedure for training subjects to change their attentional patterns and alleviate anxiety disorders (Mogg and Bradley, 2002; Mogoaşe et al., 2014; Wieckowski et al., 2019). Attentional bias training improves anxiety regulation under stress, leading to better competition performance (Ping et al., 2018). In their seminal 2012 study, McLeod et al., the dot-probe paradigm was employed to administer ABMT to patients with anxiety disorders, demonstrating that ABMT can alter the patient’s attentional bias and suppress anxiety symptoms (MacLeod and Mathews, 2012). Jia et al. (2016) found that anxious college students have an attentional bias when they experience negative emotional words, i.e., once their attention is captured by negative information, it is difficult to extricate themselves from it (Jia et al., 2016). In a competition environment, frequent changes to the athletes’ emotional states occur and high anxiety can adversely affect their performance. This is because anxiety can make athletes pay more attention to anxiety-related information, especially negative and threatening information. Although existing research confirms the improving effects of emotion regulation on athletes’ psychological state (Ji, 2023), the mechanisms by which emotion-attention interaction mechanisms affect sport performance have not yet been clarified. In particular, the role of attentional bias training in anxiety contexts on the remodeling of athletes’ attentional patterns remains a gap area in current research in sport psychology. The purpose of this study was to explore this issue and provide a targeted approach to pre-competitive attentional regulation to alleviate pre-competitive anxiety and reduce hyperfocus on negative information in order to improve athletes’ performance.

In recent years, significant research has been directed at attentional bias correction training, with studies showing that the process of attentional allocation can be altered through repeated practice (Mansell, 2004). In a study where anxious individuals were used as subjects, it was found that individuals preferentially perceived threatening information and that anxious individuals were highly sensitive to threatening information (Mathews and MacLeod, 1994). Prior research suggests that processing emotionally salient information can reinforce attentional biases toward similar emotional content in the future (Waters et al., 2009). Another study found that attentional bias of individuals may change with the stimuli (Lin, 2012). The visual detection task is the most commonly used method for assessing attentional bias in anxiety. In this task, stimuli of varying emotional content are briefly displayed on a computer screen, followed by the appearance of a small visual detector at the location where one of the emotional stimuli was presented (Cisler and Koster, 2010). The participants are required to quickly identify the direction of the detector point, with selective attention to negative stimuli being indicated when the detector appears at the location of the negative emotional stimulus. This assessment technique has been extensively confirmed in clinical and non-clinical studies investigating attentional bias toward negative stimuli (Koster et al., 2006). Research based on point detection tasks has provided empirical evidence that individuals with higher levels of anxiety are vigilant to threats (Koster et al., 2004). Although existing attentional training paradigms have focused on developing individual attentional patterns via behavioral tasks, the purpose of attentional bias correction tasks is to develop a subjects’ visual search attentional patterns by requiring subjects to identify target stimuli from multiple stimuli simultaneously and to repeatedly inhibit attention to negative stimuli during task performance, which causes changes to attentional patterns. Researchers have demonstrated that attentional bias correction training reduces subjects’ emotional anxiety about anxious events, as well as minimizes attention to threatening information (Amir et al., 2009). In this study, we hypothesized that: (1) attentional bias correction training would reduce athletes’ anxiety levels (2) attentional bias correction training would change athletes’ attentional patterns and reduce their tendency to attend to negative information (i.e., negative information attentional bias) during competition.

## Methodology

### Participants

The selection of experimental subjects was conducted from Yangzhou University, and the final number of participants was 32 high-level basketball players (national basketball level 2 or above). These athletes had extensive experience in provincial and municipal competitions and had normal visual acuity or corrective strength. In this study, 32 high-level basketball players were assigned to an experimental group (*n* = 16, 11 males and 5 females, mean age 22.30 ± 0.94 years) and a control group (*n* = 16, 10 males and 5 females, mean age 21.90 ± 1.10 years). Stratified group randomization (sequences generated by SPSS 27.0, stratified by gender) was used to ensure homogeneity between groups of pre-competition anxiety scores. Grouping information was blinded to outcome assessors, but participants were not blinded due to training task differences (emotional vs. neutral stimuli).

### Sample size calculation

The sample size required for the study was calculated using G*Power3 (Faul et al., 2009). In this study, the medium effect size was set at 0.25, the probability of one type of error *α* = 0.05, the statistical test power 1−*β* = 0.8, the level of the between-subjects variable was 2, the within-subjects level was 2 × 2 = 4, the intragroup correlation coefficient was 0.5, the sphericity test defaulted to 1, and the ratio of each training group to the control group was 1: 1. Upon calculating and combining with the previous study (Liu et al., 2017), and taking into account the 20% sample attrition rate. The sample size is expected to be at least 32 people need to be selected for the experiment. The required total sample size was 32, with 16 participants in each group, meeting the minimum requirement suggested by G*Power.

### Ethics and consent statement

All participants reported normal or corrected-to-normal vision (self-reported) and had no knowledge of the expected outcome of the study. Each participant provided written informed consent to participate and they were not paid to participate. The study was approved by The Ethics Committee of Yangzhou University School of Medicine, with project number “YXYLL-2023-115.” All rights of the participants were protected, and all experiments were performed following the 2013 Declaration of Helsinki guidelines.

### Experimental design

This study was a three-factor mixed experiment with 2 (between groups: divided into experimental and control groups) * 2 (test time: front side, back side) * 3 (mood type: positive, negative, neutral).

The within-group independent variables were mood type (positive, neutral, negative) and test time (pre-test, post-test). Dependent variables included attentional bias scores (measured experimentally via a point-probe task) and self-rated anxiety scores (anxiety situation simulation post-test). The design enables a systematic analysis of how emotional stimuli and experimental manipulations affect attentional patterns and subjective anxiety levels over time.

### Experimental process

#### Pre- and post-experiment measurements

##### Practice experiments (attention bias correction training)

**Figure 1:**
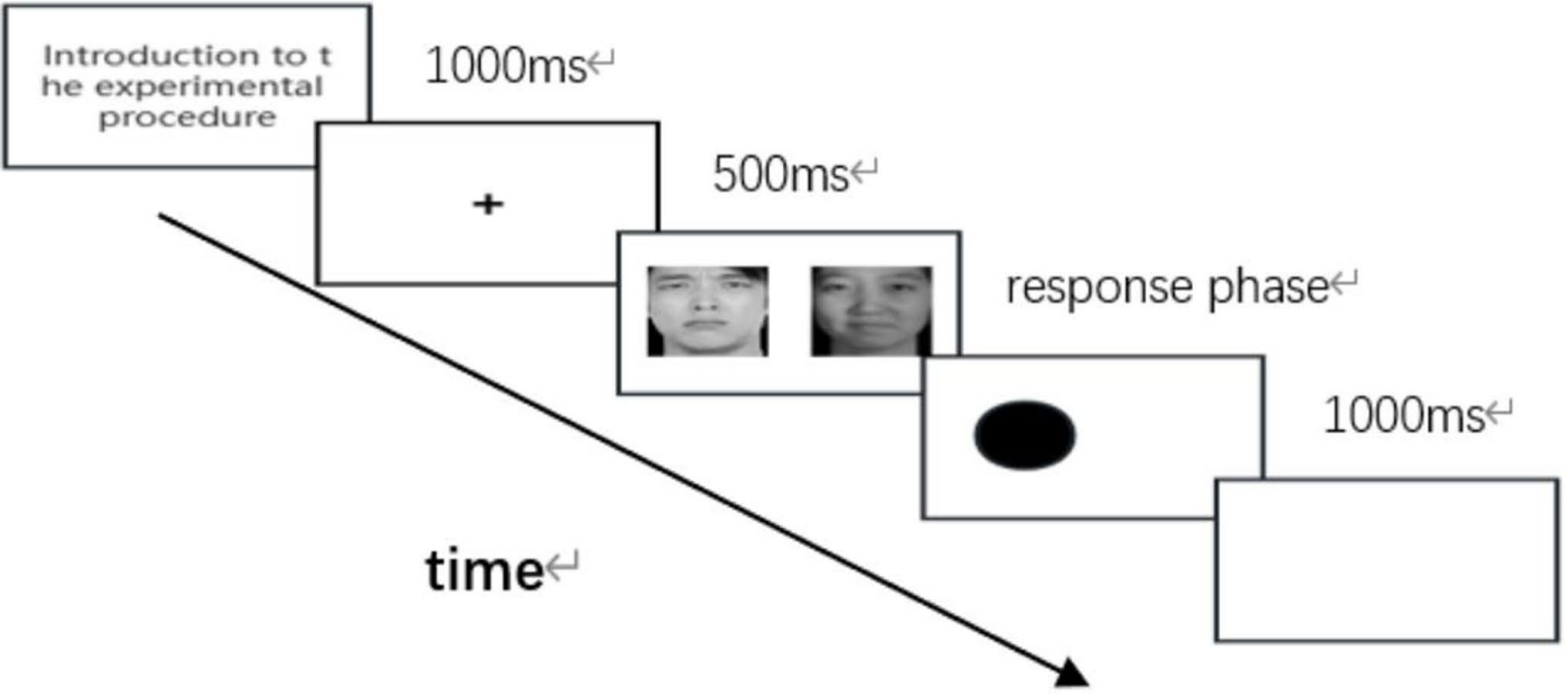
Attention bias task flowchart.

Point detection task: Before the experiment began, the participants were given a detailed explanation of the purpose, procedure, and relevant precautions of the experiment. The experiment was conducted using E-prime software with a black background. During the point detection task, a “+” symbol appeared at the center of the screen for 1000 milliseconds as a cue indicating that the stimulus was about to be triggered. Next, two photos of emotional expressions are displayed on the left and right sides, each lasting 500 milliseconds. Subsequently, the detection point appears as a white circle on one of the emotional faces. The participant must determine the location of the detection point, pressing the “F” key for the left side and the “J” key for the right side. The interval between the disappearance of the detection point and the next trial is 1000 milliseconds. The order of the photos is randomized (Figure 1).

Participants were randomly assigned to experimental or control groups using stratified randomization (by gender) in SPSS. The experimental group included 16 subjects (10 males, 6 females; age = 22.30 ± 0.94 years), and the control group comprised another 16 subjects (10 males, 6 females; age = 21.90 ± 1.10 years). Participants in both groups were trained for 4 weeks at two-day intervals. The training materials for both groups were edited and presented using E-prime software (Hakamata et al., 2010). Each training phase involved 120 trials.

**Figure 2:**
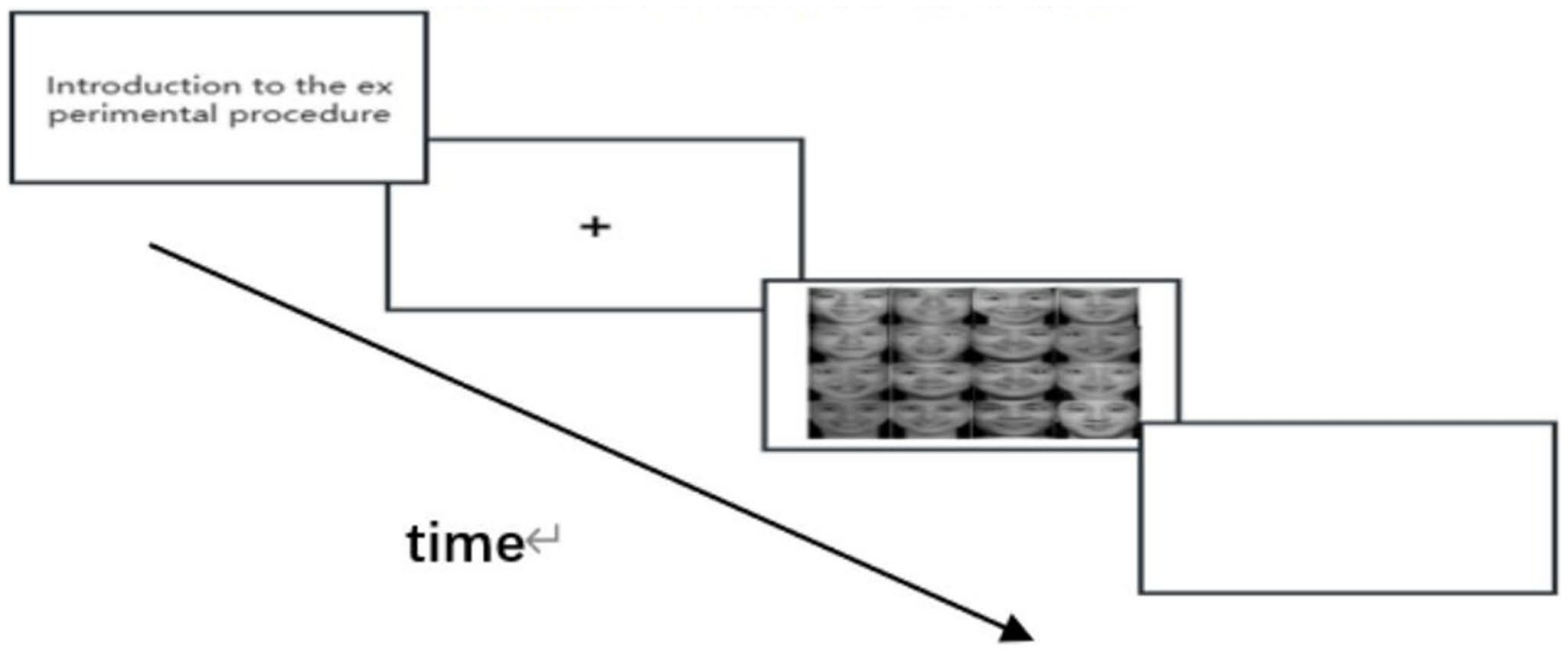
Visual search task flowchart for experimental group.

In the experimental group training, the training materials for the test subjects were 16 emotional pictures containing 15 disgusted faces and 1 happy face. The test subjects were required to quickly identify the happy face and use the mouse to click on the face (Figure 2).

**Figure 3:**
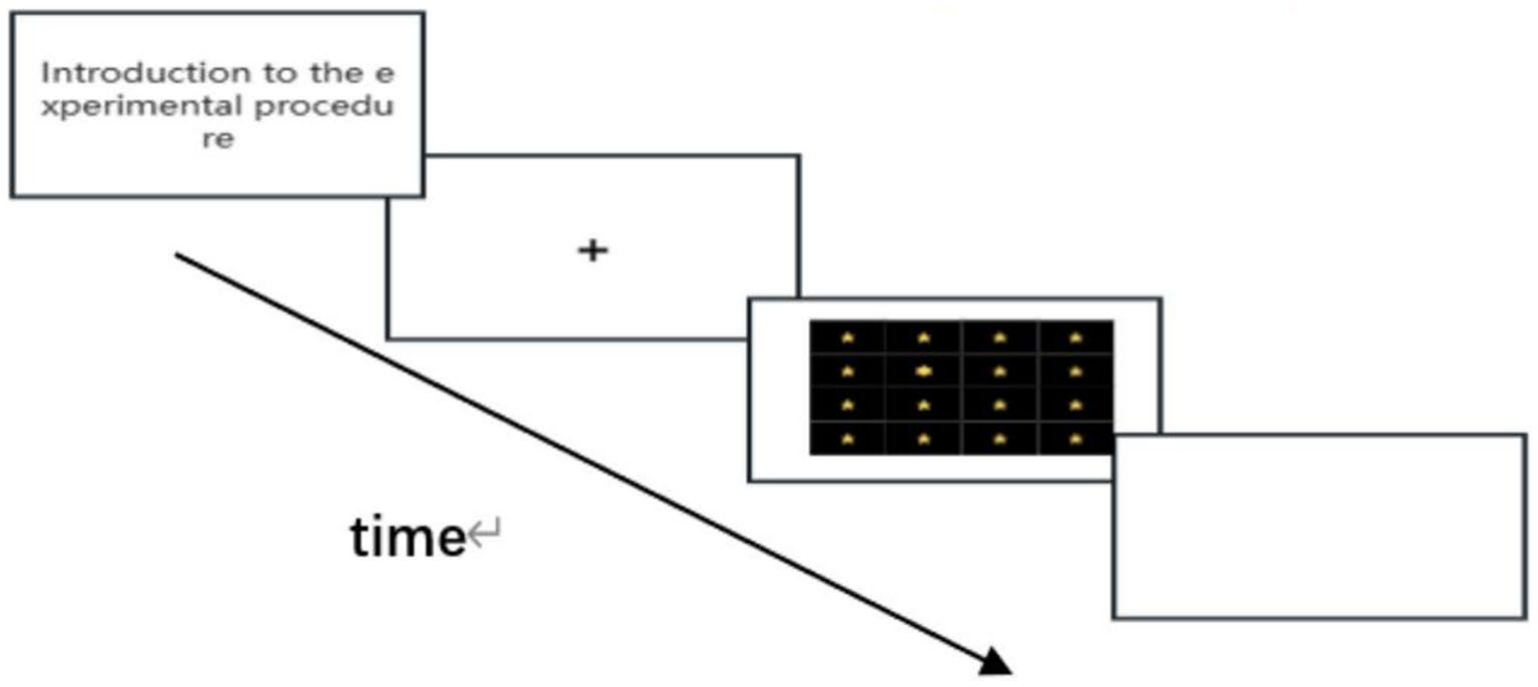
Visual search task flowchart for the control group.

In the control group training, the training materials for the test subjects were 16 star-shaped pictures containing 15 five-pointed stars and 1 six-pointed star. The test subjects were required to quickly find the five-pointed star and click on the five-pointed star using the mouse (Figure 3).

## Attention to bias testing

### Test materials

Individuals demonstrate higher response accuracy when exposed to stimuli that are consistent with their cultural background compared to when exposed to stimuli that are inconsistent with their cultural background. Given that all participants were high-level athletes with a high degree of variability in their vocabulary, a customized attentional bias modification training program based on pictures of Chinese faces from the Chinese Faces Picture Library is proposed (Wei and Zhao, 2012).

Furthermore,60 positive, neutral and negative faces were selected from the Chinese emotion picture system, and each of the 40 college students was allowed to evaluate arousal and pleasure (Bai et al., 2005). In the Chinese emotion picture system, 24 positive, 24 negative, and 48 neutral face pictures were selected as stimulus materials. With reference to the point detection paradigm, we created 24 positive-neutral, 24 negative-neutral, and 24 neutral-neutral picture pairs, which were all 260 × 300 mm in size and bmp format. The pictures and the (left–right) balancing of their presented positions, as well as the balancing of the picture positions and detection point positions were conducted using the E-prime 2.0 software (Table 1).

**Table 1:** Descriptive statistics of mood pictures (M ± SD).

Photos	Descriptive statistics of emotional pictures	
	Valence	Arousal
Positive faces	5.98 ± 0.88	5.00 ± 1.15
Negative face	2.71 ± 0.44	6.29 ± 1.23
Neutral faces	4.28 ± 0.56	3.60 ± 0.48

### Anxiety scenario simulation

A similar test design was employed in response to the methods commonly used to simulate stress (Moore et al., 2012). The experiment was performed before the competition, and subjects were informed that the score of this test will affect their final overall performance. If the test score was below a certain score line, the subjected were asked to complete an additional 3,200-meter running task. In this experiment, the subjects were divided into 10 groups, each with two members, and the group’s overall performance directly affected each member’s evaluation. In order to enhance the objectivity and credibility of the test, selected experts and team members watched live as observers so that the players could feel the same pressure of being watched as in a real competition. This setup allowed the athletes to experience the tension of a competition in advance of the test, making the results of the study more relevant to the actual situation.

### Test procedure

The stimuli were presented using the E-prime 2.0 software, with the experimental screen set to black and the experimental stimuli set to white to reduce eyestrain for individuals. At the start of the dot-probe task, subjects were introduced to the program used in the experiment before testing. At the beginning of the experiment, a “+” symbol appeared in the center of the screen for 1,000 ms to indicate that the stimulus was about to appear. Next, two pictures of emotional faces appeared on either side of the “+” symbol for 500 ms. Next, a white circular probe appeared at one of the locations of the previously appeared emotional face pictures. Participants were instructed to identify and click on the location of the dot based on what they saw. To determine the location of the dot, press the “F” key on the keyboard if it is on the left side, or press the “J” key on the keyboard if it is on the right side. Once the key reaction was triggered, the detection point disappeared immediately, and the next round of testing was started (Figure 1). The interval between each test was 1,000 ms, and to avoid the formation of subjects’ response habits, the presentation order and presentation time of each set of picture pairs were randomized to ensure that the subjects achieved their best presentation within 3 rounds.

### Note the bias score algorithm

The reaction times to the target stimuli in the vicinity of the emotional faces were automatically recorded using the E-Prime 2.0 software. Attentional bias scores were obtained by calculating the reaction time when the probe appeared in the position of the paired neutral face minus the reaction time when the probe appeared in the position of the negative or positive face. An attentional bias score > 0 indicated the presence of an attentional bias toward emotional faces. If it was = 0, attentional bias was deemed absent. and if it was < 0, avoidance of emotional faces (a bias in favor of neutral faces) was considered present (Liu et al., 2017).

### Experimental tools

The Pre-Competition Emotions Scale-T (Tension for, 2001) consists of four subscales of 32 questions, each consisting of 8 questions (Zhang, 2000), all scored on a 5-point Likert scale (1 = not at all, 5 = fully). The self-confidence subscale measures athletes’ positive beliefs about their abilities (e.g., “I believe I can do well in the competition”); the personal failure anxiety subscale assesses concerns about underperformance (e.g., “I am afraid of underperforming in the competition”); the somatic anxiety subscale examines pre-competition physiological reactions (e.g., “I often feel sweaty palms or shivering before the competition”); and the social desirability anxiety subscale focuses on social appraisal anxieties (e.g., “I worry about disappointing people who support me”). The internal consistency reliability (Cronbach’s alpha coefficient) of each subscale ranged from 0.78 to 0.86 (Liu et al., 2017), and the retest reliability (2-week interval) ranged from 0.72 to 0.81, which indicated that the scale had a good level of reliability and was able to measure the athletes’ pre-game emotional state stably and reliably.

### Data processing

SPSS 27.0 was used for statistical analysis. False response trials (e.g., timeout, false key presses) and extreme values beyond ±3 standard deviations were excluded from the data preprocessing stage. Separate repeated-measures ANOVAs were conducted for self-assessed anxiety levels and attentional bias scores to examine the effects of time (pre-test/post-test) or experimental condition and to analyze the interaction between the two. Where necessary, Pearson correlations were used to test the association between anxiety and attentional bias, supplemented by *post hoc* comparisons with Bonferroni correction. Statistical significance was set at *p* < 0.05, Greenhouse–Geisser correction was used when the sphericity assumption was not met, and *η^2^_p_* was reported as an effect size indicator.

## Results

### Comparison of self-assessed anxiety level

A repeated—measures analysis of variance was conducted with the group (experimental group, control group) and test time (pretest, posttest) as independent variables and the self-rated pre-competition anxiety scale as the dependent variable (Table 2). The main effect of test time was significant [*F*(1, 32) = 48.879, *p* < 0.001, η^2^ₚ = 0.449], indicating that the test time had a significant impact on the anxiety level; the main effect of the group was significant [*F*(1, 32) = 5.078, *p* < 0.05, η^2^ₚ = 0.078], indicating a significant difference between the experimental group and the control group; the interaction effect between test time and group was significant [*F*(1, 32) = 19.794, *p* < 0.001, η^2^ₚ = 0.248], indicating that the change in anxiety level varied by group, and the change in anxiety level at different test time points was more significant in the experimental group.

**Table 2:** Self-rated anxiety scores before/after training (M ± SD).

Groups	Pre-game anxiety scores			
	Pre-testing	Post-test	*P*	*η*^2^ₚ
Experimental group	44.00 ± 2.280	37.81 ± 1.377**	0.001	0.752
Control subjects	42.81 ± 2.536	41.44 ± 2.279	0.155	0.071

* *p* < 0.05, ** *p* < 0.01, compared with pre-training.

**Figure 4:**
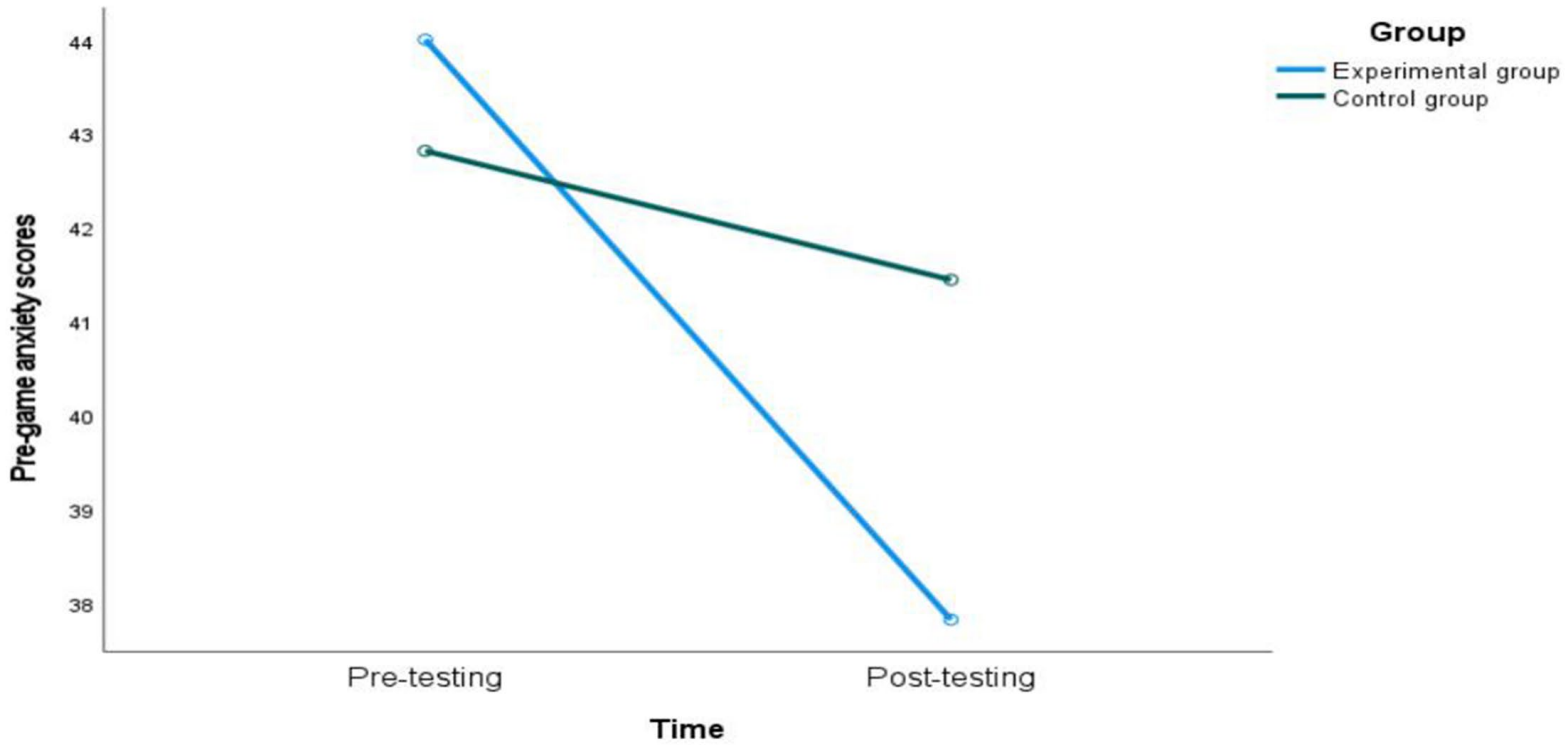
Graph of pre-game anxiety score changes.

Due to the significant interaction effect between the group and test time, further simple—effect analysis was performed (Figure 4). In the experimental group, the posttest pre-competition anxiety score (37.81 ± 1.377) was significantly lower than the pretest score (44.00 ± 2.280), and the difference was statistically significant (*p* < 0.001). In the control group, there was no significant difference between the posttest pre-competition anxiety score (41.44 ± 2.279) and the pretest score (42.81 ± 2.536) (*p* > 0.05). The pre-competition anxiety score of the experimental group decreased significantly after training, indicating that attention—bias training had a positive effect on relieving pre-competition anxiety.

### Comparison of attentional bias scores

A repeated—measures analysis of variance was conducted with the group (experimental group, control group) and test time (pretest, posttest) as independent variables and the attention—bias scores obtained from completing the dot—probe task in an anxiety—inducing situation as the dependent variable (Table 3). The main effect of the group was significant [*F*(1, 30) = 45.945, *p* < 0.001, η^2^ₚ = 0.434], indicating a significant difference in attention—bias scores between the experimental group and the control group; the main effect of test time was significant [*F*(1, 30) = 26.098, *p* < 0.001, η^2^ₚ = 0.303], indicating that the test time had a significant impact on attention—bias scores; the main effect of emotion type was significant [*F*(1, 30) = 176.231, *p* < 0.001, η^2^ₚ = 0.746], indicating that the emotion type significantly affected attention bias; the interaction effect between the group and test time was significant [*F*(1, 30) = 48.277, *p* < 0.001, η^2^ₚ = 0.446], indicating that there were significant differences in the changes of attention—bias scores between the experimental group and the control group at different test times; the interaction effect between test time and emotion type was significant [*F*(1, 30) = 16.352, *p* < 0.001, η^2^ₚ = 0.241], indicating that the interaction between test time and emotion type significantly affected attention bias; the interaction effect between the group and emotion type was not significant [*F*(1, 30) = 3.953, *p* > 0.05, η^2^ₚ = 0.062], indicating that the group had no significant effect on emotion type; the three – way interaction among the group, test time, and attention—bias scores was not significant [*F*(1, 30) = 2.780, *p* > 0.05, η^2^ₚ = 0.044], indicating that the complex interaction among the group, test time, and emotion type did not reach a significant level.

**Table 3:** Comparison of attention bias scores before/after training (M ± SD).

Groups	Positive-medium emotional faces		Negative-medium emotional faces	
	Pre-testing	Post-test	Pre-testing	Post-test
Experimental group	6.05 ± 2.85	4.41 ± 2.09	1.99 ± 0.57	−4.51 ± 0.26**
Control subjects	5.85 ± 3.44	7.49 ± 3.44	2.07 ± 0.79	1.68 ± 0.70

* *p* < 0.05, ** *p* < 0.01, compared with pre-training.

**Figure 5:**
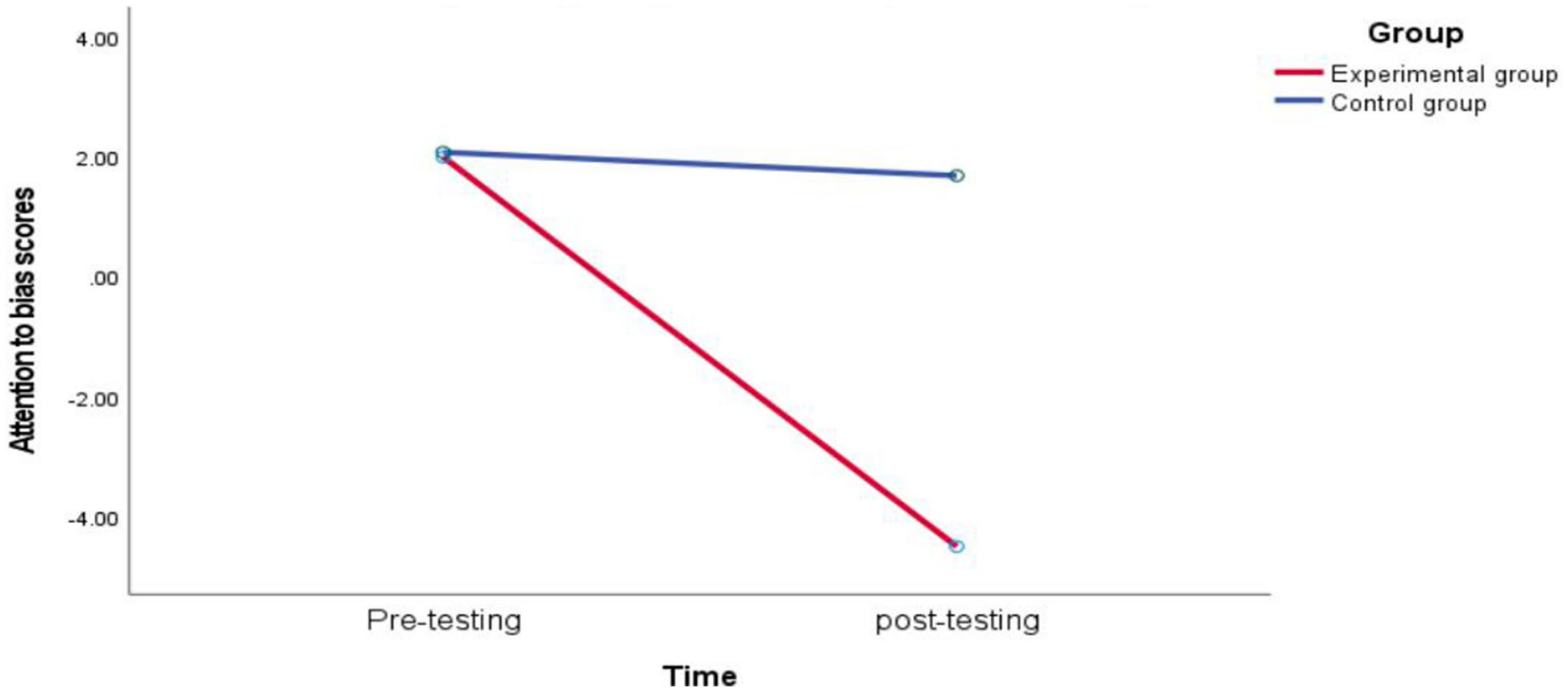
Plot of change in attentional bias scores for negative emotional faces.

Further simple—effect analysis showed (Figure 5). In the negative—neutral emotional face task, the difference between the pre-training (1.99 ± 0.57) and post-training (−4.51 ± 0.26) scores of the experimental group was significant (*p* < 0.001), while the difference between the pre-training (2.07 ± 0.79) and post-training (1.68 ± 0.70) scores of the control group was not significant (*p* > 0.05). In the positive—neutral emotional face task, there was no significant difference between the pre-training (6.05 ± 2.85) and post-training (4.41 ± 2.09) scores of the experimental group (*p* > 0.05), and there was also no significant difference between the pre-training (5.85 ± 3.44) and post-training (7.49 ± 3.44) scores of the control group (*p* > 0.05).

When the experimental group performed the dot—probe task under anxiety conditions, before training, there was a significant difference between the attention—bias score for negative emotional states and zero (*p* < 0.05), with a score of 1.99 > 0, indicating that the experimental group had an attention bias towards negative emotional states before training. After training, there was also a significant difference between the attention—bias score for negative emotional states and zero (*p* < 0.05), with a score of −4.51 < 0. This indicates that after training, the attention—bias score of the experimental group towards negative emotional faces decreased significantly, and they were more inclined to positive faces. After training, the experimental group’s attention bias towards negative emotional faces decreased significantly, and they were more inclined to pay attention to positive faces. Attention—bias training successfully and effectively changed the experimental group’s attention—allocation pattern for negative emotional stimuli, making them more inclined to avoid negative emotional information.

## Discussion

The purpose of this study was to investigate whether attentional bias modification training (ABMT) reduces pre-competition anxiety and attentional bias to negative information in high-level athletes. We hypothesized that (1) ABMT would reduce self-reported anxiety levels and (2) attenuate attentional bias to negative stimuli. The results of the study supported these hypotheses, demonstrating significant reductions in both metrics after the intervention.

In this study, we designed the attentional bias correction training to develop the visual attention patterns of athletes in which they were asked to search for a target among multiple simultaneous stimuli that require repeated inhibition of negative stimuli and to change their visual attention patterns by searching for negative stimuli. The 4-week (every 2 days) attentional bias correction training revealed reduced attentional bias for negative information and pre-game anxiety in athletes under anxiety conditions at the end of the training.

The dot-probe paradigm, which was first-proposed in a study on visuospatial attention, is applied to examine attentional bias (Koster et al., 2005). Notably, attention to emotional stimuli can be evaluated using emotional facial expressions as stimuli when performing a dot-probing task (Brosch et al., 2008). For comparison, we calculated difference in the response times to target stimuli from the pictures of positive and negative emotional faces and the response times to target stimuli from pictures of neutral emotional faces, i.e., it was converted into an attentional bias score (response times when the probe dots appeared in the position of a paired neutral face minus response times when the probe dots appeared in the position of a negative or positive face). The results showed that the pre-training attentional bias score of the experimental group was 4.99 > 0 and the post-training attentional bias score was −5.99 < 0. Therefore, we inferred that the pre-training reaction time to stimuli next to negative emotional faces was relatively short. After training, the participants demonstrated significantly longer reaction time to stimuli in this area. This suggests that prior to the training, the subjects paid more attention to the location of the negative emotional face during the attentional bias correction training, indicating a pre-existing attentional bias toward negative information. However, after the training, the athletes paid less attention to the location of negative emotional faces and showed attentional avoidance of neutral faces.

These results suggest that the mechanism of attentional bias to prioritize the processing of negative stimuli is rooted in the threat warning system developed during human evolution, a cognitive trait that promotes survival adaptations by enhancing sensitivity to potentially dangerous information (Peng and Zhou, 2005). During species adaptation, the human nervous system prioritizes the processing of negative signals with an early warning function, and this rapid recognition mechanism has evolved and been optimized over time to enable organisms to be able to avoid existential threats in a timely manner, thereby increasing the likelihood of survival (Curby and Collins, 2024). In addition, psychological stressors (e.g., anxiety and stress) exacerbate attentional bias, prompting individuals to hyperfocus on negative information in potential threats (Pellicane and Ciesla, 2024). This psychological state is particularly evident in high-pressure environments such as competitive sport, where it allows athletes to gain an advantage in processing negative information, which in turn has a significant impact on their ability to make mental adjustments and overall athletic performance (Pond et al., 2023).

Attentional bias training reduces selective attention to negative stimuli, which can reduce mood swings and optimize mental load distribution, thus contributing to the stability of in-competition performance. This attentional conditioning strategy facilitates the establishment of positive mental orientations, which enables athletes to better maintain game focus and effectively filter out distractions in the environment. At the same time, attentional bias training not only strengthens emotional regulation mechanisms, but also promotes the development of mental toughness in stressful situations, so that athletes are in an optimal psychological state during competitive confrontation.

In this study, anxiety scenarios in which the timing of spectators, competition, punishment, and testing affect motor performance were simulated using common methods. Meanwhile, real-time observation by experts and teammates was introduced to augment social evaluation pressure, replicating the state of being in the spotlight in a real game by administering the test prior to the game (Moore et al., 2012). This environmental variable increased tension in the scenario, competition enhanced motivation to succeed, and punishment increased subjects’ anxiety about the test. By doing the test before competition, we expose the subjects to anxiety, which helps to minimize anxiety (Liu, 2009). The “emotional congruence” effect suggests that subjects in a particular emotional state will prioritize the processing of information that is congruent with that particular emotional state. Since stressful situations tend to increase anxiety level in subjects, they are more likely to pay attention to anxiety-related information selectively, especially negative and threatening information, i.e., they have an attentional bias towards negative information (Liu and Qian, 2005). The results of this study indicate that after a four-week (every 2 days) of attentional bias correction training, the subjects showed reduced levels of pre-game anxiety and attentional bias toward negative information when completing on a point detection task under anxious state. This suggests that attentional bias correction training can affect emotion regulation.

In the attentional bias correction training, subjects are forced to search for positive stimuli by repeatedly suppressing attention to negative stimuli. This potentially changes the subject’s attentional pattern, reducing the attentional bias towards negative information and attentional bias in anxiety situations. By minimizing attention to negative information, its presence in the participants’ focus is shortened, thereby reducing the activation of negative emotions and the perception of anxiety. This effect contributes to lower anxiety levels, diminished attention to negative and threatening stimuli, and further reduced attention to negative information. This implies that attentional bias correction training can moderate the mood by altering the subjects’ attentional patterns to reduce the attention bias toward negative information. In addition, attention to threatening information was decreased when mood was improved (anxiety level was reduced).

The present study not only verified the effectiveness of Attention Bias Modification Training (ABMT) in alleviating pre-competition anxiety and improving attentional bias in athletes, but also has important implications at the theoretical and practical levels. At the theoretical level, the results of the study deepen our understanding of the emotion-attention interaction mechanism and provide new evidence for cognitive interventions in sport psychology; at the practical level, the training program can be directly applied to the athlete psychological training system, especially for individualized interventions for athletes with a high tendency to anxiety, and the experimental paradigm and methodology adopted in this study also provide a template for psychological interventions in other high-pressure contexts. At the same time, the experimental paradigm and methodology adopted in this study also provide a template for other psychological interventions in high-pressure situations.

## Conclusion

In summary, this study highlights the dual benefits of ABMT in reducing pre-competition anxiety and mitigating attentional bias toward negative information. By bridging theory and practice, it opens new avenues for cognitive interventions in sports psychology and beyond. Future research should explore broader applications and long-term outcomes to maximize the potential of this training approach.

The study’s primary limitation is its small sample size, which may affect statistical power and external validity. Although our power analysis justified the sample, replication with larger groups is critical. Additionally, all participants were high-level basketball athletes; results may not generalize to other sports or amateur athletes.

## Data Availability

The raw data supporting the conclusions of this article will be made available by the authors, without undue reservation.
